# Imaging features of high-grade astrocytoma with piloid features: A single center case series

**DOI:** 10.1177/19714009251395689

**Published:** 2025-11-15

**Authors:** Heba Al Qudah, Jackson D Hamilton, Maria A Gubbiotti, Ahmed Msherghi, Hamza A. Salim, Sahar Alizada, Ho-Ling Liu, Vinodh A. Kumar, Max Wintermark, Rami W. Eldaya

**Affiliations:** 1 Department of Neuroradiology, 4002 The University of Texas MD Anderson Cancer Center, Houston, TX, USA; 2 Division of Pathology and Laboratory Medicine, The University of Texas MD Anderson Cancer Center, Houston, TX, USA; 3 Department of Imaging Physics, 4002 The University of Texas MD Anderson Cancer Center, Houston, TX, USA

**Keywords:** High-grade astrocytoma with piloid features, novel imaging features, focal diffusion restriction, perilesional spiculated, finger-like enhancement, neurofibromatosis type 1, ependymal enhancement

## Abstract

High-grade astrocytoma with piloid features (HGAP) is a recently described IDH-wildtype tumor with limited literature describing its imaging features. We present our institution’s experience and the largest imaging-focused cohort of HGAP reported to date, offering a comprehensive analysis of MRI features of seventeen intracranial and one spinal HGAP, while introducing novel imaging features that have not been previously described. These include focal areas of diffusion restriction, surrounding spiculated, finger-like enhancement and increased perfusion metrics. Additionally, we highlight imaging features of HGAP arising in patients with Neurofibromatosis Type 1, such as intraventricular tumor spread, unusual temporal lobe location and multifocal presentation.

## Introduction

High-grade astrocytoma with piloid features (HGAP) is one of the newly described tumors in the fifth edition of the World Health Organization (WHO) classification of the Central Nervous System (CNS) tumors, which further emphasizes and adopts new advances in molecular diagnostics of CNS neoplasms in line with Consortium to Inform Molecular and Practical Approaches to CNS Tumor Taxonomy.^1–7^ HGAP is characterized by a distinct DNA methylation profile,^8^ that differentiates it from typical childhood pilocytic astrocytoma, a frequently overlapping entity on histological analysis.^9^ Few HGAP cases have been reported in the literature and they have not demonstrated specific imaging features, making it challenging to include HGAP in the imaging differential diagnosis. In this case series, we provide a detailed characterization of the diverse imaging presentations of seventeen intracranial cases and one spinal case confirmed by pathology and DNA methylation profiling, with particular emphasis on tumor locations, the variety of enhancement patterns observed, and a discussion of advanced imaging when available at the time of presentation. We further assess the imaging features of HGAP in patients with Neurofibromatosis Type 1.

## Case series

### Patient cohort

This study was approved by the Institutional Review Board at The University of Texas MD Anderson Cancer Center, with a waiver of informed consent due to its retrospective design. This study was conducted and reported in accordance with the CARE (Case Report) guidelines. We recruited a total of 28 consecutive patients with a potential pathological diagnosis of HGAP between July 2019 and February 2025. Molecular analysis and DNA methylation profiling were performed for each patient in accordance with the WHO 5th edition criteria for central nervous system (CNS) neoplasms to establish the definitive diagnosis of high-grade astrocytoma with piloid features (HGAP). Seven patients were excluded due to unavailable tissue for confirmatory DNA methylation profiling, and an additional four were excluded due to lack of initial tumor imaging. Ultimately, a total of eighteen cases (one spinal case and seventeen intracranial cases) met the inclusion criteria, which required a confirmed diagnosis based on molecular analysis and DNA methylation, imaging documentation, and the availability of pre-treatment imaging. A detailed summary of the selection criteria is summarized in the Patient Selection Flowchart.


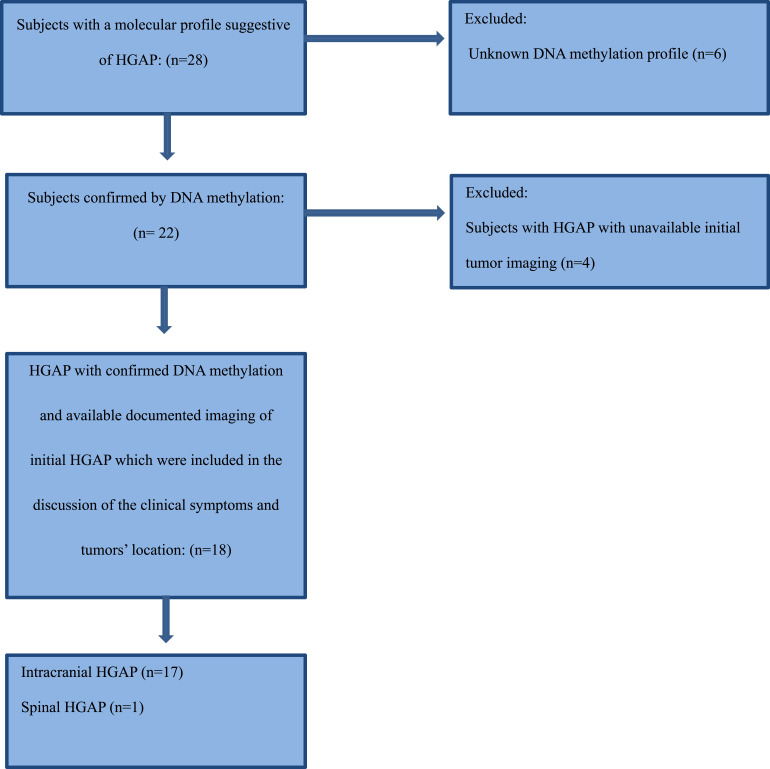


Ten patients included in this cohort were previously reported in a separate pathology-neurosurgery case series.^10^ However, that publication did not focus on imaging features, and the imaging data presented in the current manuscript were not included in the previous report.

### Data review

Clinical data were extracted from electronic medical records and included age at the time of diagnosis, biological sex, presenting symptoms, and neurofibromatosis 1 (NF1) status when available.

Imaging data were evaluated for tumor location and size in three dimensions (anteroposterior, transverse, and craniocaudal), as well as signal characteristics on T1-weighted, T2-weighted, FLAIR, DWI, and ADC maps, and gradient sequences (SWI/GRE). The pattern of post-contrast enhancement was assessed, along with additional imaging features such as the presence of a mass effect, hydrocephalus, cystic components, and microhemorrhage. We also reported the presence of multifocal disease when more than one lesion was identified. Additionally, advanced imaging techniques, including dynamic susceptibility contrast (DSC), dynamic contrast-enhanced imaging (DCE), arterial spin labeling (ASL), magnetic resonance spectroscopy (MRS), and PET/CT, were analyzed whenever available.

All imaging assessments were conducted in consensus by a fellowship-trained neuroradiology attending (R.E.), with 6 years of dedicated neuroradiology experience, and a second-year neuroradiology fellow (H.A.), with 2 years of dedicated neuroradiology experience. Pathology data extraction, molecular profiling, and DNA methylation analysis were reviewed by a board-certified neuropathologist (M.G.).

### Statistical analysis

Categorical variables were summarized as frequencies with percentages. Continuous variables were summarized as medians with interquartile ranges (IQR) and compared using the Wilcoxon rank sum exact test. R statistical software (version 4.3.0, R Project for Statistical Computing) and Rstudio statistical software (version 2023.03.0 + 386, Rstudio) were used for statistical analyses.

## Results

### Clinical presentations and demographic features

Eighteen patients met the eligibility criteria for this study. The median age at diagnosis was 42.5 years (interquartile range, 34-57 years). Nine patients were female, and nine were male. The most common presenting symptoms were dizziness (50%), followed by headache (44%).

One patient had a remote history of medulloblastoma diagnosed ten years earlier at the same site where HGAP later developed. Unfortunately, the primary tumor was diagnosed and treated outside our institution, and tissue samples were not available at the time of the new HGAP diagnosis to confirm whether it was originally HGAP.

Clinical and imaging features of NF1 were present in five patients. Two of the NF1 cases had a remote history of an ependymoma or a pilocytic astrocytoma at sites distant from the HGAP tumor.

A detailed summary of the clinical features is provided in Table 1.

**Table 1.** Clinical features.

Characteristic (number of patients)	Findings (number of patients)
Age at diagnosis in years (*n* = 18)	Median (IQR): 42.5 (34-57)
Sex (*n* = 18)	Female: 9
	Male: 9
Presenting symptoms (*n* = 16)^a^	Dizziness: 8
	Headache: 7
	Nausea and vomiting: 5
	Gait instability: 3
	Blurred vision: 4
	Numbness: 2
	Neck pain: 2
	Hearing disturbance: 1
	Loss of consciousness: 2
	Memory issues/loss: 3
	Falling down: 1
	Urinary retention: 1
	Facial nerve palsy: 1
Other pertinent findings	Clinical/imaging features of NF1: 5/18

^a^Initial presenting symptoms were unavailable in 2 cases (2/18). Most patients presented with more than one symptom.

### Imaging features

The average tumor dimensions were 3.10 cm (IQR 1.50–4.00) anteroposteriorly, 2.90 cm (IQR 1.60-3.90) transversely, and 2.45 cm (IQR 2.20–4.00) craniocaudally. There was frequent involvement of multiple brain regions, and for simplicity, these locations were divided into the posterior fossa, diencephalon, spinal cord, and temporal lobe (Figures 1-13).

**Figure 1.**
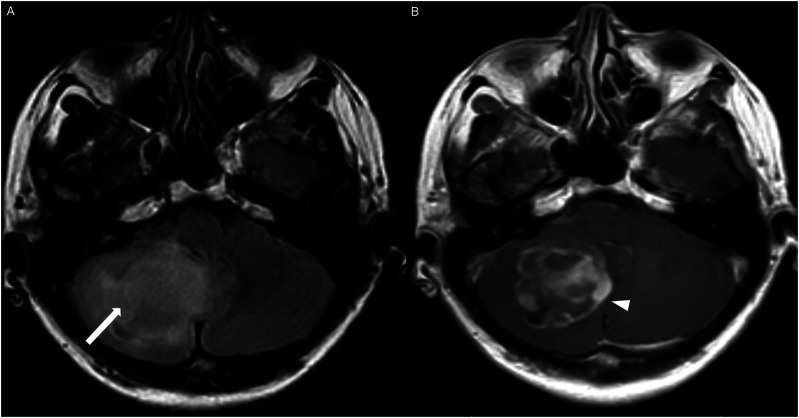
Axial FLAIR (A) and axial post-contrast T1 weighted images (B), show a FLAIR hyperintense mass involving the right cerebellum (A, arrow), demonstrating heterogeneous enhancement (B, arrowhead). There is surrounding edema, mass effect and partial effacement of the fourth ventricle.

**Figure 2.**
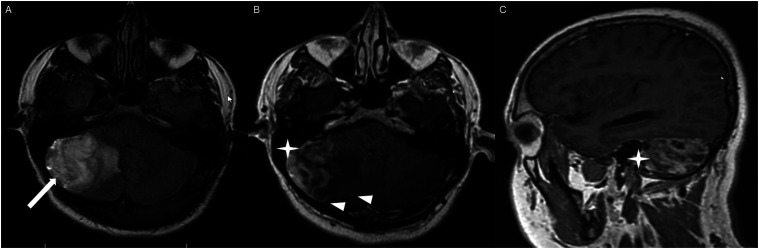
Axial FLAIR (A), axial post-contrast T1 weighted image (B), sagittal post-contrast T1 weighted image (C) show a FLAIR hyperintense mass (A) in the lateral aspect of the right cerebellum (A, arrow), demonstrating heterogeneous and gyriform post-contrast enhancement (B-C, star). There is perilesional spiculated, finger-like enhancement (B, arrowhead).

**Figure 3.**
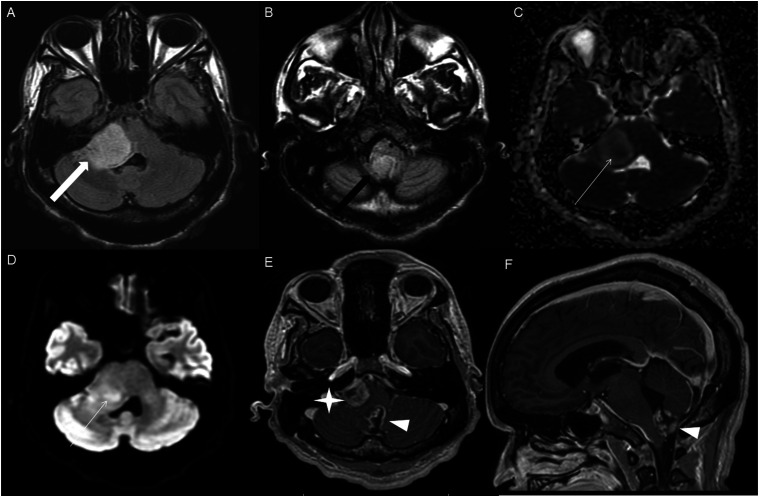
Axial FLAIR (A-B), DWI (C), ADC map (D), axial post-contrast T1 weighted image (E) and sagittal post-contrast T1 weighted image (F) of an NF1 patient show two concurrent FLAIR hyperintense tumors in different locations; the right middle cerebellar peduncle (A, thick white arrow), demonstrating heterogeneous post-contrast enhancement (E, star) and a focal area of diffusion restriction (C-D, thin white arrows) and the second tumor involving dorsal medulla and cerebellar tonsils (B, thick black arrow), with extension into the fourth ventricle, and showing peripheral, irregular enhancement (F, arrowhead).

**Figure 4.**
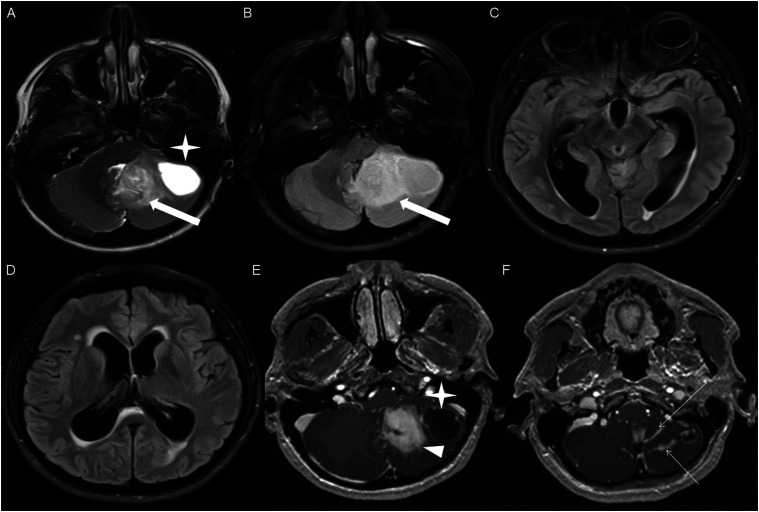
Axial T2 weighted image (A), axial FLAIR (B-D) and axial post-contrast T1 weighted image (E-F), show a T2/FLAIR hyperintense mass involving the left cerebellum (A-B, thick white arrow), with mixed cystic (A, star) and solid enhancing components (E, arrowhead). Inferiorly, there is surrounding spiculated, finger-like enhancement (F, thin arrows). There is mass effect and effacement of the fourth ventricle, with obstructive hydrocephalus and transependymal CSF flow.

**Figure 5.**
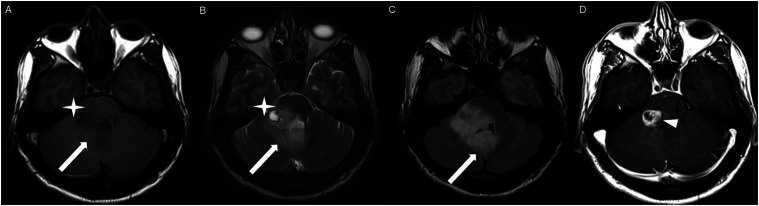
Axial T1-weighted image (A), axial T2-weighted image (B), axial FLAIR (C) and axial post-contrast T1 image (D), show a T1 hypointense, T2/FLAIR hyperintense mass (A-C, thick white arrow) involving the right middle cerebellar peduncle, right side of the pons and midbrain and cerebellar vermis, with mixed non-enhancing and nodular-enhancing components (D, arrowhead). Focal cystic changes are also seen on T2-weighted image (B, star).

**Figure 6.**
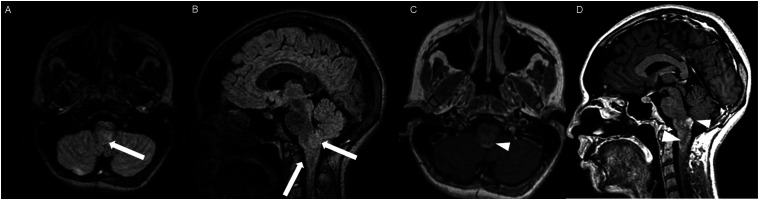
Axial FLAIR (A), sagittal FLAIR (B), axial post-contrast T1 weighted image (C) and sagittal post-contrast T1 weighted image (D) in an NF1 patient with a remote history of resected right cerebellar pilocytic astrocytoma, show an expansible FLAIR hyperintense mass involving the posterior medulla and cervicomedullary junction (A-B, thick white arrows) with heterogeneous striated and nodular enhancement (C-D, arrowhead).

**Figure 7.**
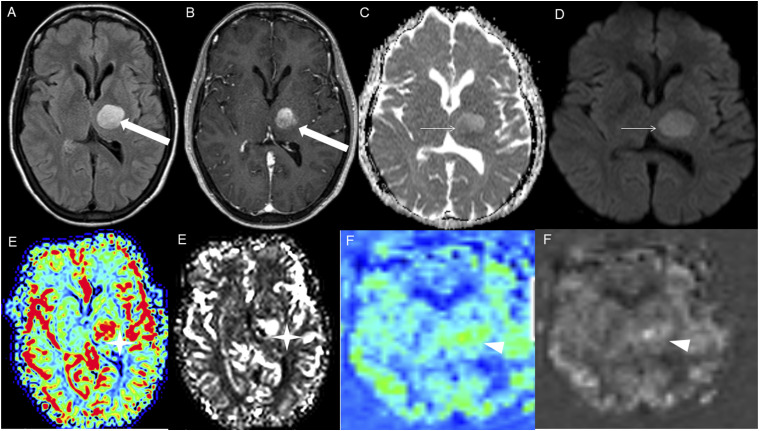
Axial FLAIR (A), post-contrast T1 weighted image (B), DWI (C), ADC map (D), dynamic susceptibility contrast DSC (E) and arterial spin labeling perfusion ASL (F), show a FLAIR hyperintense mass involving the left thalamus (A, thick white arrow) with heterogeneous post-contrast enhancement (B, thick white arrows). There is focal area of diffusion restriction medially on DWI/ADC map (C-D, thin arrows). There is elevated relative cerebral blood volume on DSC (E-star) and increased cerebral blood flow on ASL (F-arrowhead).

**Figure 8.**
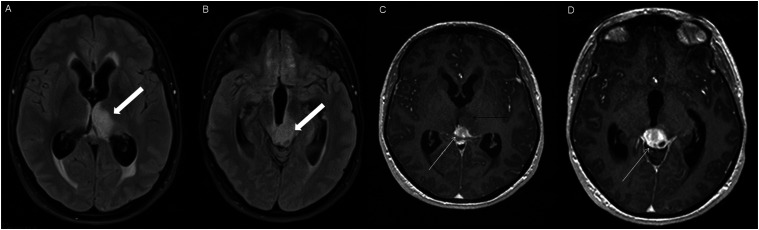
Axial FLAIR (A-B) and post-contrast T1 weighted images (C-D), show a FLAIR hyperintense mass involving the left thalamus and quadrigeminal plate (A-B, thick white arrows), with mixed non-enhancing (C, thin black arrow) and nodular-enhancement components (C-D, thin white arrows). There is mass effect and obstruction of the third ventricle with obstructive hydrocephalus and transependymal CSF flow.

**Figure 9.**
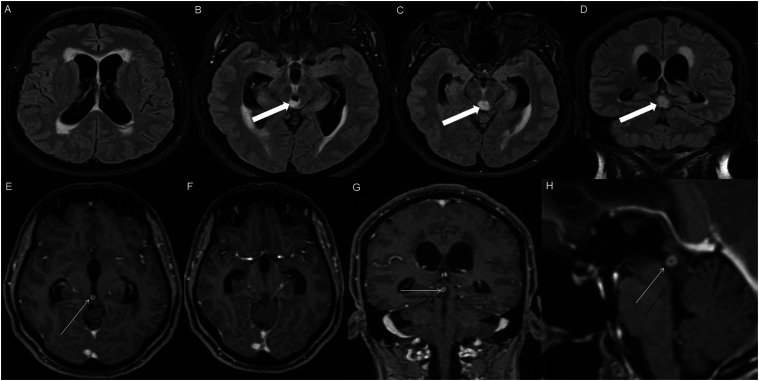
Axial FLAIR (A-C), coronal FLAIR (D), axial post-contrast T1 weighted images (E-F), coronal post-contrast T1 weighted image (G) and sagittal post-contrast T1 weighted image (H), show a FLAIR hyperintense mass involving the quadrigeminal plate and periaqueductal area (B-D, thick white arrows), with mixed non-enhancing (F-H, thin black arrow) and ring-enhancement components (E,G-H, thin white arrow). There is mass effect and obstructive hydrocephalus, with transependymal CSF flow.

**Figure 10.**
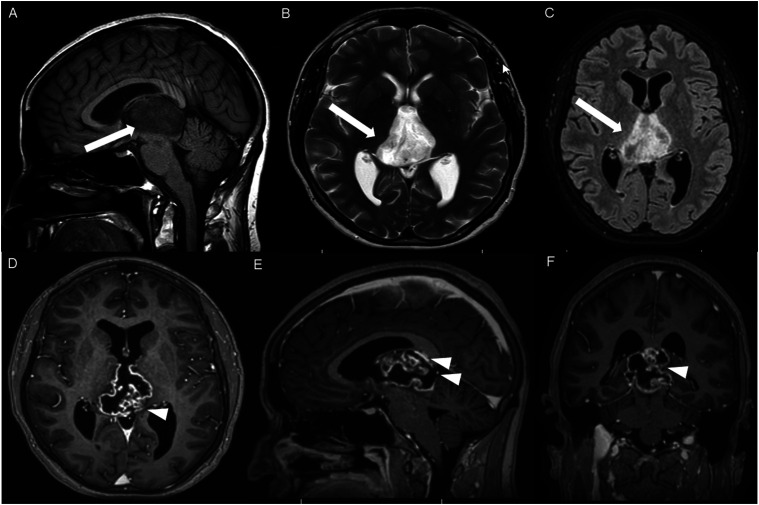
Sagittal T1 weighted image (A), axial T2 weighted image (B), axial FLAIR (C), axial post-contrast T1 weighted image (D), sagittal post-contrast T1 weighted image (E) and coronal post-contrast T1 weighted image (F) show a T1 hypointense, T2/FLAIR hyperintense mass involving the third ventricle and medial thalami (A-C, arrows) with peripheral irregular enhancement (D-F, arrowhead). There is obstruction at the level of the foramen of Monro, with hydrocephalus.

**Figure 11.**
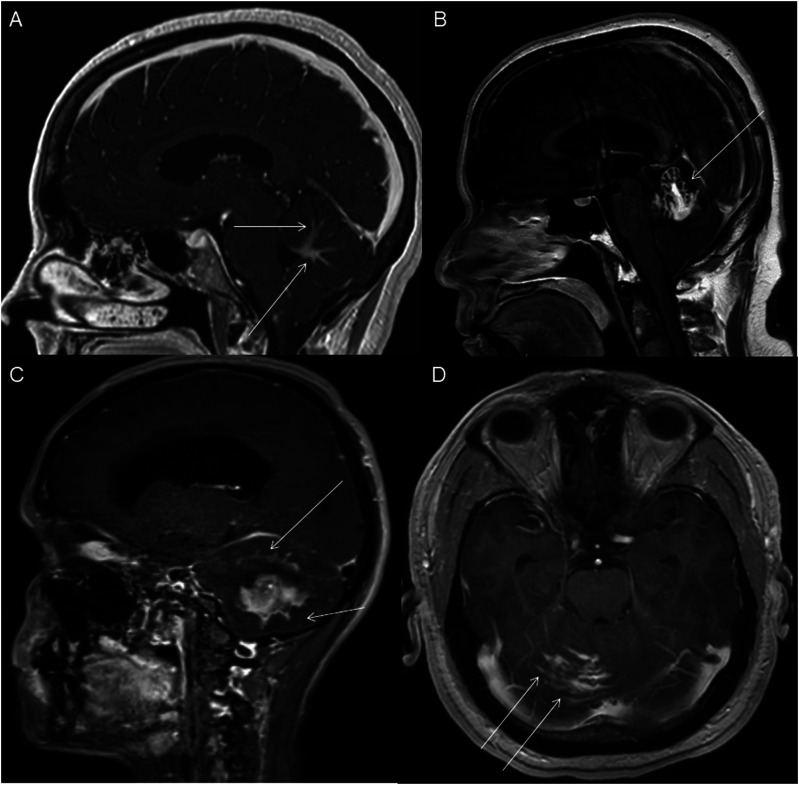
Sagittal post-contrast T1 weighted image (A-C) and axial post-contrast T1 weighted image (D) for four different patients demonstrate perilesional spiculated, finger-like enhancement (thin arrows).

**Figure 12.**
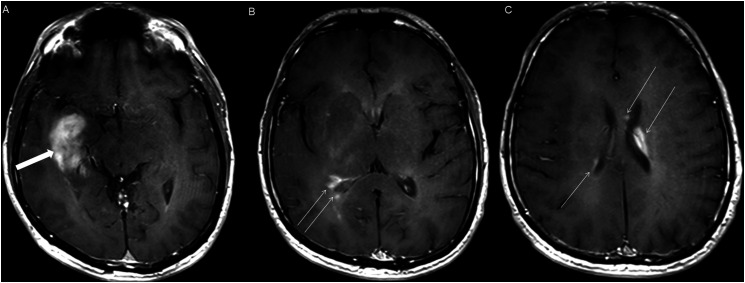
Axial post-contrast T1 weighted images (A-C) for an NF1 patient show a heterogeneously enhancing mass involving the right hippocampus and temporal lobe (A, thick arrow) with ependymal enhancement suggestive of intraventricular seeding (B-C, thin arrows).

**Figure 13.**
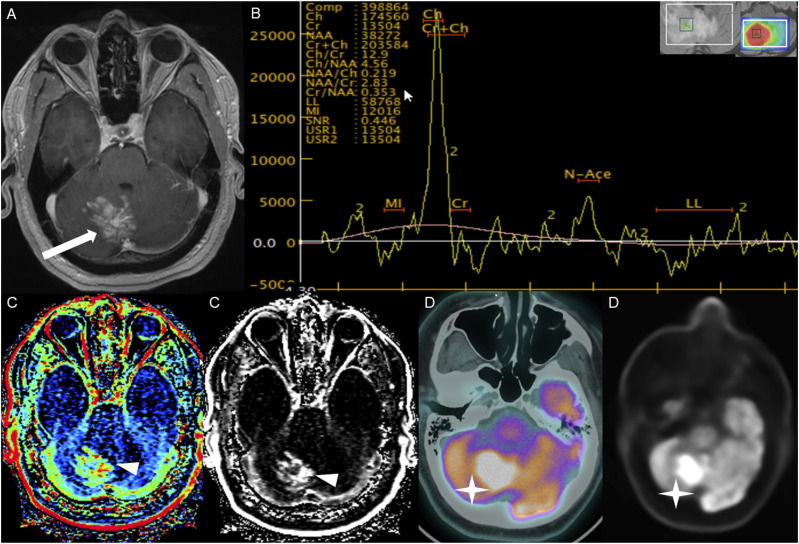
Axial post-contrast T1 weighted image (A), Multi-voxel MR Spectroscopy (B), Dynamic Contrast-Enhanced (DCE) imaging (C), and FDG PET-CT (D) show a heterogeneously enhancing mass in the right cerebellum (A, arrow), which demonstrates increased choline-to-creatine ratio with values reaching up to 4.56 and decreased NAA on multi-voxel MR Spectroscopy (B). There is increased capillary permeability on DCE (C, arrowhead) and increased metabolic activity on FDG PET/CT (D, star).

HGAP was most commonly encountered in the posterior fossa, accounting for 10 of the 18 cases (Figures 1-6). In 9 of the 10 posterior fossa cases, there was involvement of and/or extension to the cerebellum. The diencephalon was affected in five cases (Figures 7-10).

The hippocampi and temporal lobes were involved in two cases (Figure 12). One of the two temporal lobe cases demonstrated extension into the basal ganglia and thalamus. Additionally, one tumor originated in the spinal cord.

All intracranial lesions were T1 hypointense and T2/FLAIR hyperintense relative to gray matter. Mass effect was observed in 16 of the 17 intracranial cases. Hydrocephalus was noted in 8 of the 17 intracranial cases. A cystic component was present in 8 of the 17 intracranial cases (Figures 4 and 5). Additionally, foci of susceptibility signal loss were identified in 3 of the 15 cases where gradient sequences were available.

DWI and ADC were available at the time of diagnosis for 16 cases; focal diffusion restriction was observed in 8/16 cases (Figures 3 and 7). The median ADC value was 776 × 10^−3^ (IQR 646-809) mm^2^/s in the cases with focal diffusion restriction compared to 1109 × 10^−3^ (IQR 1012-1312) mm^2^/s in cases without diffusion restriction (P-value = 0.002).

A detailed description of the imaging features is summarized in Table 2.

**Table 2.** Tumor locations and imaging features.

Characteristics (number of patients)	Findings
Tumor dimensions in cm (*n* = 18)	Anteroposterior
	Median (IQR): 3.10 (1.50-4.00)
	Transverse
	Median (IQR): 2.90 (1.60-3.90)
	Craniocaudal
	Median (IQR): 2.45 (2.20-4.00)
ADC (*n* = 16)^a^	Focal diffusion restriction (*n* = 8)
	Median (IQR): 776 (646-809) ×10^−3^ mm^2^/s
	No diffusion restriction (*n* = 8)
	Median (IQR): 1,109 (1,012–1312) ×10^−3^ mm^2^/s
	P^1^ 0.002 (wilcoxon rank sum exact test)

^a^ADC/DWI were available at the time of diagnosis for 16 cases.

HGAP cases demonstrated variable and inconsistent appearances and enhancement patterns (Figures 1-10). The most frequent pattern was heterogeneous enhancement, which was observed in five cases of posterior fossa tumors (Figure 1) and two cases of diencephalic tumors (Figure 7).

Peripheral, irregularly enhancing masses were observed in one posterior fossa case (Figure 3) and one diencephalic case (Figure 10).

Three cases demonstrated a mixed enhancement pattern, with both enhancing and non-enhancing tumor components. Among these, the enhancing areas were nodular in one posterior fossa case (Figure 5) and in one diencephalic case (Figure 8), while ring enhancement was observed in one diencephalic mass (Figure 9).

Other enhancing patterns, each observed in a single case of posterior fossa HGAP, included a gyriform enhancement pattern (Figure 2), a mixed solid and cystic mass with a solid enhancing component (Figure 4), and a striated and nodular-enhancement pattern (Figure 6).

The presence of surrounding spiculated, finger-like enhancement was observed in 12 of the 17 intracranial cases and was exclusively seen in the posterior fossa and temporal lobe cases (Figure 11).

### Specific imaging characteristics in cases with imaging or clinical features of neurofibromatosis type 1

Two of the five NF1 patients developed tumors of the temporal lobes and hippocampi (Figure 12(A)), which to our knowledge, represent the first HGAP cases occurring outside the posterior fossa, diencephalon, and/or spinal cord.^1,10^

Ependymal enhancement and intraventricular seeding (Figure 12(B)-(C)) were exclusively observed in cases with imaging or clinical features of neurofibromatosis type 1, occurring in all five NF1 cases (5/5).

Two concurrent HGAP tumors in different locations in one of the five NF 1 cases (Figure 3), involving the right middle cerebellar peduncle and the dorsal medulla/cerebellar tonsils, with extension into the fourth ventricle.

### Advanced imaging findings

Advanced imaging was available at the initial presentation for a limited number of cases in our series. Dynamic susceptibility contrast (DSC) MRI was available in three cases, and elevated relative cerebral blood volume^11,12^ (rCBV) observed in two of the three cases, showing a tumor-to-white matter (WM) ratio of 3.2 (Figure 7(E)). Intermediately elevated rCBV of tumor/WM ratio of 1.6 was noted in one case.

Arterial spin labeling (ASL) was available in six cases at the time of HGAP diagnosis, with increased cerebral blood flow observed in five of the six cases (Figure 7(F)).

Multi-voxel MR spectroscopy with intermediate TE was available for two cases at the time of HGAP diagnosis. Both cases demonstrated an increased choline-to-creatine ratio (Cho/Cr) with values greater than 2 and depressed N-acetylaspartate (NAA) levels (Figure 13(B)).

Dynamic contrast enhanced (DCE) MRI was available in four cases, all of which demonstrated significantly elevated capillary permeability (Figure 13(C)).

Additionally, HGAP tumors demonstrated increased metabolic activity on PET-CT in all three intracranial cases (3/3). Increased FDG uptake was observed in two cases (Figure 13(D)), and increased DOTATATE uptake was noted in one case.

### Spinal HGAP

The single spinal HGAP case presented as a T1 hypointense, T2 hyperintense intramedullary mass spanning a long segment of the spinal cord from C2 to T1, with a predilection for the dorsal cord and exhibiting patchy and heterogeneous enhancement. Associated cord expansion was noted. FDG PET-CT demonstrated increased metabolic activity.

## Discussion

High-grade astrocytoma with piloid features (HGAP) is generally considered a circumscribed astrocytic glioma with a unique methylation profile.^1^ The WHO has not yet assigned a specific grade for HGAP but it is likely to be of grade 3 or 4^1^. Histologically, HGAP tumors display variable features ranging from piloid cytology to frank anaplasia, as seen in glioblastoma.^4^ The most common genetic abnormalities are *CDKN2A/B* deletion, *MAPK* pathway alterations (typically involving *NF1*, *BRAF*, and *FGFR1*), and *ATRX* mutation or loss of expression.^1^ HGAP is associated with Neurofibromatosis Type 1(NF1),^1,13^ and evaluation of the DNA methylation profile should be considered to rule out HGAP when there is a histologically high-grade glioma arising in the setting of NF1.^9^ DNA methylation profiling should be considered when there is a cerebellar glial neoplasm that does not fit neatly into another WHO diagnostic category, especially in the setting of ATRX loss and/or *CDKN2A* homozygous deletion.^9^

The median age at diagnosis was 42.5 years in our case series, which is similar to the reported median age of 41.5 years.^1^ All patients were symptomatic at presentation, with dizziness and headache being the most common presenting features. This aligns with the literature, where headache is reported as the most common presenting symptom.^14^ The presence of hydrocephalus in approximately 47% of our cohort at diagnosis may explain the high frequency of these symptoms.

The most common location was the posterior fossa, accounting for 55.6% of cases, with the cerebellum involved in 50% of total cases. Tumors were T1 hypointense and T2 hyperintense, consistent with previous reports.^1,14^

Focal diffusion restriction was observed in approximately 50% of the cases. The diffusion restriction of HGAP tumors has not been detailed in prior studies.^14–16^

To date, no single dominant enhancement pattern for HGAP has been reported in the literature,^14^ and our case series demonstrates multiple enhancement patterns. The most common pattern was a predominantly enhancing mass with heterogeneous enhancement, present in 50% of the posterior fossa tumors and 40% of the diencephalon tumors.

Although HGAP has traditionally been described as a circumscribed glioma, we observed spiculated, finger-like perilesional enhancement in 71% of intracranial cases. To our knowledge, this enhancing spiculation has not been reported in prior case series. This feature may serve as a potential marker for HGAP diagnosis. However, studies with larger patient populations are needed to validate this finding further. Furthermore, correlation with surgical and pathological reports is needed to better assess the clinical significance and pathophysiology of this finding.

Prior studies have not described the imaging features of HGAP in patients with Neurofibromatosis Type 1^14^. A unique imaging feature in our NF cohort was ependymal enhancement, suggestive of intraventricular tumor spread, which was present in all five cases. This may represent a distinct pattern of tumor dissemination in NF1 patients and could serve as a radiological marker for the diagnosis of HGAP in these patients. It may also help distinguish HGAP from pilocytic astrocytoma in NF1 patients. However, larger studies are needed to further assess the prevalence and diagnostic significance of this finding in NF1 patients with HGAP.

Two of the five NF1 patients had HGAP that uniquely involved the hippocampus and temporal lobe, a location not previously described for HGAP.^14,15^ Furthermore, one of the NF1 patients presented with two concurrent HGAPs, which to our knowledge, represents the first documented case of multifocal HGAPs.

HGAP tumors showed [18F]-FDG avidity, consistent with a previously reported single case of [18F]-FDG avidity.^14^ One HGAP case demonstrated increased DOTATATE uptake, a feature not previously reported in the literature.^14^ HGAP demonstrated increases in capillary permeability on dynamic contrast enhanced (DCE) imaging, increased relative cerebral blood volume (rCBV) on dynamic susceptibility contrast (DSC) and increased cerebral blood flow on arterial spin labeling (ASL). MR spectroscopy revealed increased choline-to-creatine ratio (Cho/Cr) and depressed N-acetylaspartate (NAA) levels. These features have not been previously described in the literature.^14^ The increased perfusion metrics, along with the presence of diffusion restriction may play a significant role in differentiating HGAP from pilocytic astrocytoma (PA), particularly because these tumors are common in the posterior fossa and both have been reported to be associated with NF1.^17^ PA does not typically exhibit increased perfusion or diffusion restriction.^17^

HGAP shares many imaging features with the extremely rare infratentorial glioblastoma, which accounts for less than 1% of all glioblastomas,^18^ including heterogeneous enhancement, non-enhancing tumor regions, focal diffusion restriction, elevated perfusion metrics,^19^ and tumor signature on spectroscopy.^20^ Furthermore, both HGAP and glioblastoma are IDH wildtype,^20^ which poses a diagnostic challenge for radiologists and pathologists. The most reliable method for differentiating these entities is DNA methylation profiling.

This study has several limitations, including its retrospective design and the lack of detailed treatment documentation in some referred cases, largely due to the absence of standardized management protocols.^2^ However, these limitations are offset by robust inclusion criteria and the fact that HGAP is an emerging entity with few cases discussed in the radiology literature. Furthermore, this case series represents the largest imaging-focused cohort of HGAP reported to date, presenting unique features that have never been reported, including the focal diffusion restriction, the surrounding spiculated finger-like enhancement, and increased perfusion metrics. Additionally we reported unique features of HGAP arising in NF1 patients, such as intraventricular tumor spread, unusual temporal lobe location, and multifocal presentation.

## Conclusion

HGAP is a circumscribed astrocytic glioma characterized by a distinct DNA methylation profile. It often arises in the posterior fossa, diencephalon, and spinal cord and has been reported in association with NF1. Radiologically, HGAP commonly demonstrates heterogeneous enhancement, focal diffusion restriction, elevated perfusion, and a characteristic tumor signature on MR spectroscopy. A potentially unique imaging feature is spiculated perilesional enhancement. In NF1 patients, ependymal enhancement suggestive of intraventricular tumor spread was consistently observed, with rare cases showing multifocal involvement or unusual temporal lobe location.

## Appendix

### Abbreviations

MRI: Magnetic Resonance Imaging
FLAIR: Fluid Attenuation Inversion Recovery
HGAP: High-grade astrocytoma with piloid features
IQR: Interquartile range
PA: Pilocytic astrocytoma
DCE: Dynamic contrast-enhanced
ASL: Arterial spine labeling
DSC: Dynamic susceptibility contrast
WHO: World Health Organization
CNS: Central Nervous System
rCBV: Relative Cerebral Blood Volume
DWI: Diffusion-Weighted Imaging
ADC: Apparent Diffusion Coefficient
NF1: Neurofibromatosis Type 1
MRS: Magnetic Resonance Spectroscopy
Cho/Cr: Choline-to-Creatine ratio
NAA: N-acetylaspartate.
